# Ultra-High Sensitivity Terahertz Microstructured Fiber Biosensor for Diabetes Mellitus and Coronary Heart Disease Marker Detection

**DOI:** 10.3390/s23042020

**Published:** 2023-02-10

**Authors:** Jia Xue, Yani Zhang, Zhe Guang, Ting Miao, Zohaib Ali, Dun Qiao, Yiming Yao, Kexin Wu, Lei Zhou, Cheng Meng, Nigel Copner

**Affiliations:** 1 Department of Physics, School of Arts & Sciences, Shaanxi University of Science & Technology, Xi’an 710021, China; 2 School of Physics, Georgia Institute of Technology, 837 State Street NW, Atlanta, GA 30332, USA; 3 Nano-Optoelectronics Research Laboratory, Department of Physics, University of Agriculture Faisalabad, Faisalabad 38040, Pakistan; 4 Faculty of Computing, Engineering and Science, University of South Wales, Pontypridd CF37 1DL, UK; 5 School of Electrical and Control Engineering, Shaanxi University of Science & Technology, Xi’an 710021, China

**Keywords:** diabetes mellitus, coronary heart disease, microstructured fiber, terahertz, effective material loss, sensitivity

## Abstract

Diabetes Mellitus (DM) and Coronary Heart Disease (CHD) are among top causes of patient health issues and fatalities in many countries. At present, terahertz biosensors have been widely used to detect chronic diseases because of their accurate detection, fast operation, flexible design and easy fabrication. In this paper, a Zeonex-based microstructured fiber (MSF) biosensor is proposed for detecting DM and CHD markers by adopting a terahertz time-domain spectroscopy system. A suspended hollow-core structure with a square core and a hexagonal cladding is used, which enhances the interaction of terahertz waves with targeted markers and reduces the loss. This work focuses on simulating the transmission performance of the proposed MSF sensor by using a finite element method and incorporating a perfectly matched layer as the absorption boundary. The simulation results show that this MSF biosensor exhibits an ultra-high relative sensitivity, especially up to 100.35% at 2.2THz, when detecting DM and CHD markers. Furthermore, for different concentrations of disease markers, the MSF exhibits significant differences in effective material loss, which can effectively improve clinical diagnostic accuracy and clearly distinguish the extent of the disease. This MSF biosensor is simple to fabricate by 3D printing and extrusion technologies, and is expected to provide a convenient and capable tool for rapid biomedical diagnosis.

## 1. Introduction

Diabetes mellitus (DM) is a metabolic disorder due to defective secretion of insulin or its impaired biological action in the human body, resulting in hyperglycemia or triggering kidney and nerve dysfunctions [1,2,3]. Coronary heart disease (CHD, also known as coronary atherosclerosis) is a blockage of arteries caused by abnormal blood lipids and high blood sugar in the human body, which eventually leads to myocardial ischemia and even myocardial necrosis [4,5,6]. Because of these severe effects, there is a critical need to develop an efficient and rapid detection technique for DM and CHD. Traditional techniques for detecting CHD include coronary angiography [7] and coronary CT imaging [7,8], and for detecting DM include fasting plasma glucose (FPG) test and oral glucose tolerance test (OGTT) [3]. Coronary arteriography requires the injection of contrast agent into an aorta and the development of an X-ray image, which involves a complex procedure with many associated risks and complications, such as spasm, X-ray radiation hazards, and sensitization. Intravenous glucose measurement is a one-time test, and such monitoring can be expensive and technically demanding, which will increase the financial and time burden of patients. In recent years, terahertz (THz) microstructured fiber (MSF) biosensing, a new method for blood component detection, has attracted research attention due to its high accuracy, fast speed, and compact device size [9,10].

MSFs have been widely used in biochemical sensing based on the properties that they are inherently sensitive to changes in refractive index and/or effective material loss due to the fiber structures. For example, use of MSFs has been reported for temperature measurement [11,12], gas monitoring [13], cancer cell detection [14,15], chemical identification [9,16,17], and blood composition detection [18,19]. Compared with other types of MSFs, hollow-core MSFs are often preferred, as they provide larger contact areas between the THz wave and the analytes, and are also convenient to manufacture [9,20]. By adjusting geometric parameters, such as core diameters and pore shapes/arrangements, important optical properties including the relative sensitivity can be effectively changed. The finite element method (FEM) is usually used to simulate the optical performance, including MSF modal distribution and transmission characteristics under different designs. Optical fiber structures can be optimized until satisfactory performance is achieved.

Compared with traditional electromagnetic waves, the THz wave has been widely used in biochemical sensing because of its low energy, transient absorption, fingerprint region and water absorption properties [17]. Currently, among terahertz sensing technologies, terahertz time-domain spectroscopy (THz-TDS) [21,22], terahertz surface plasmon resonance (SPR) sensors [23,24], and terahertz surface plasmon polariton (SPP) interferometry sensors [25,26] are very popular among researchers. In particular, the application of THz-TDS, especially in tumor and other disease detection, has attracted much attention, as an emerging nondestructive detection technique. In recent years, various MSF geometries have been proposed to obtain better optical sensing performance as well as to reduce fabrication difficulties. Md. Saiful Islam et al. (2018) suggested an MSF sensor with a Kagome-shaped cladding with a porous core to detect water, ethanol and benzene [16]. This intricate fiber structure exhibits relative sensitivity of 85.7%, a near-zero flat dispersion of 0.47 ± 0.27 ps/THz/cm and a low confinement loss (CL) at the operating frequency band around 1.6 THz. The authors also reported a structure of an elliptical stomatal array core surrounded by a porous cladding based on the Zeonex material [27]. Their results show that the MSF achieves birefringence of 0.086 with moderate CL of 3.8 × 10^−9^ cm^−1^. In 2021, Md. Jayed Bin Murshed Leon et al. designed an MSF with silica as the substrate material for detecting human mucosa and glucose [28]. Results show that the best sensitivity for the analyzed material reached 47.59% and CL reached 10^−5^ cm^−1^. Comparing the results using Zeonex and silica, it can be seen that a proper selection of substrate material is one key factor to provide excellent MSF performance. In 2019, Md. Ahasan Habib et al. presented a new rectangular hollow-core MSF to detect chemical analytes [9]. This improved structure exhibited higher relative sensitivity and lower CL, and can also be relatively easily fabricated. In a more recent paper in 2020, Etu Podder et al. designed a rectangularly stacked MSF sensor based on amorphous polyolefin, which achieved relative sensitivity of 94.38% and low CL of 2.75 × 10^−13^ cm^−1^ for red blood cells at 1.8 THz [29]. However, this work left out the study of a very important parameter, the effective material loss (EML). Subsequently, Yanan Wang et al. proposed a spider-web type fiber end face structure using the same amorphous polyolefin as substrate material [30], which achieved low EML of 0.058 cm^−1^, but a relatively high CL of about 3.22 × 10^−6^ cm^−1^. Based on all the above results, it is clear that, to achieve satisfactory biosensor performance, a proper combination of substrate material and fiber structural design is critical and thus desired.

In this paper, we present an easy-to-fabricate, extremely high-sensitivity, and low-loss MSF biosensor for detecting DM and CHD marker concentrations in a broad frequency range of 0.6~2.4 THz. The MSF has a suspended-core, which ensures the performance and is simple to fabricate. Our result exhibits an ultra-high value of relative sensitivity (100.35%) for standard levels of triglycerides at 2.2 THz, and CL is maintained at an ultra-low level of about 10^−13^ cm^−1^, which is more excellent than previously reported results. Moreover, simulation results of the proposed design reveal that high power fraction and numerical aperture (NA) can be simultaneously achieved, which offers a great and convenient-to-use design for biomedical sensing applications.

## 2. Design Approach and Theoretical Derivations

### 2.1. Design and Configurations of MSF

**Figure 1:**
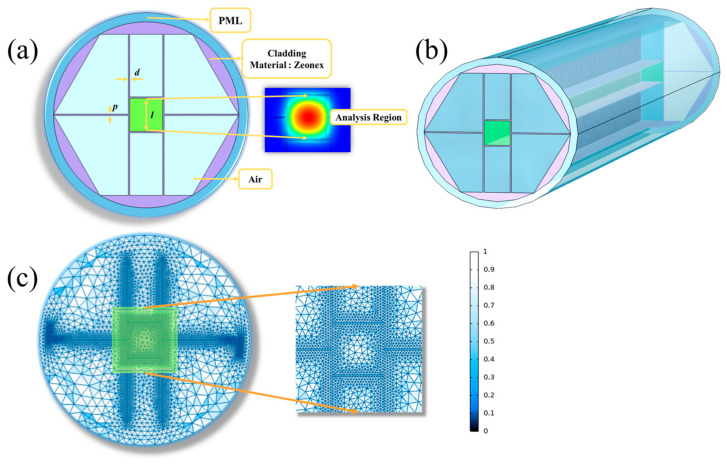
(**a**) Two-dimensional cross section diagram and local magnification diagram of the proposed MSF sensor. (**b**) Three-dimensional perspective of the MSF sensor. (**c**) Meshing diagram of the MSF cross-section and an enlarged view of the local details.

Figure 1 represents the cross-section of our designed MSF with a suspended hollow-core structure, which works excellently over a broad THz band and can be manufactured flexibly. The fiber core consists of a square with side length *l*. Meanwhile, four supporting arms extend from the fiber core outward, dividing the hexagonal cladding into six parts. The thickness of the vertical support arm is denoted as *d* and the thickness of the horizontal arm as *p*. These arms provide support for the MSF structure, which helps minimize the fiber deformation during manufacturing. Generally, a large surface area in the core increases the contact area between the THz wave and the analytes, thus improving the efficiency of the fiber sensor. In the design, the parameters are set as *l* = 500 μm, *d* = 20 μm, *p* = 30 μm. To analyze the transmission characteristics of the proposed MSF, several numerical simulation methods are applicable, such as the equivalent refractive index method, the plane wave expansion method, and FEM. Among them, FEM has become the most widely used method due to its high accuracy, versatility, and adaptability to complex structures. The essence of FEM is to (1) discretize the continuous solution domain into a finite number of non-overlapping cells, and (2) represent the unknown field solution by the obtained approximated functions in each cell. Figure 1c shows a schematic diagram of the mesh cell partitioning, and an enlarged view of the MSF cross-section. The color of the mesh cell indicates the quality of the mesh division: the lighter the color, the higher the mesh division quality, and the more likely the field will converge to an accurate realistic solution. Boundary conditions are introduced to numerically solve the problem, and then the field function and the required performance parameters are obtained for the whole simulated domain. To simulate the realistic case and achieve convergence, a special dielectric layer with a thickness of 10% of the fiber radius is set at the fiber periphery, so that THz waves leaking into the cladding can pass through the interface and enter a perfect matched layer (PML) without reflection [14,31]. Here, the radius of the fiber is 1400 μm and the PML is 140 μm in thickness.

For the fiber substrate material, we choose Zeonex, which has unique advantages of very low material loss, low water absorption, good chemical stability, low density, high temperature resistance, and a constant refractive index of about 1.5258 in the THz band compared to other polymeric materials [32,33]. Therefore, Zeonex fibers can be minimally affected by environmental factors and the material remains stable over a broad THz band, which makes Zeonex an ideal material for THz MSF sensors.

**Figure 2:**
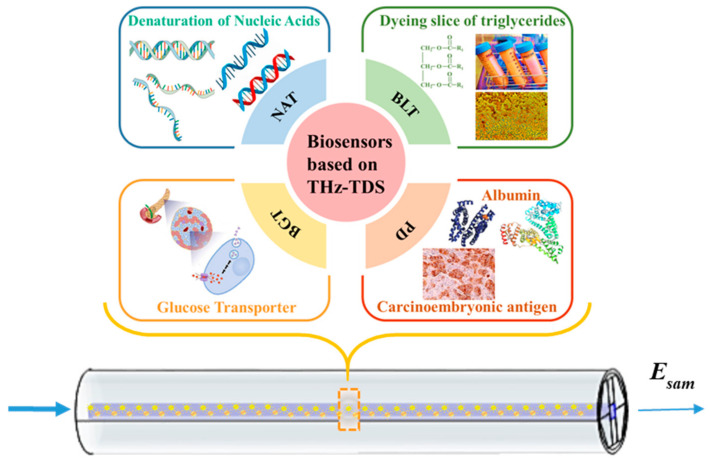
Schematic diagram of filling biological samples into the MSF sensor (NAT: Nucleic acid testing, BLT: Blood lipid testing, PD: Protein detection, BGT: Blood glucose testing). *E_sam_* represents the electrical field with sample information, at an attenuated level compared to the input due to absorption.

Currently, terahertz-based MSF biosensors have been reported for blood component detection and cancer cell detection. Biomarkers in blood cover a very wide range of biochemical substances, among which the most typical biomarkers include glucose, triglycerides, protein molecules, and nucleic acid molecules, as shown in Figure 2. CHD markers are serum solutions containing triglycerides, which are usually inhaled into the fibrils using capillary phenomena. DM markers are tablets made from large molecules such as proteins and glucose in the blood, which require grinding of sample tablets into fine particles before filling them into the core. The THz wave is in full contact with the substance to be measured in the core, and the disease markers are detected by receiving THz signals carrying information about the sample. For the length of terahertz MSF, 50 mm~100 mm is usually set as appropriate. If the fiber length is too low, the number of terahertz wave oscillations inside the fiber is too few to form a stable mode field distribution, and at the same time, it will enhance the difficulty of experimental operation; if the MSF length is too long, the MSF biosensor may face problems such as output signal attenuation, sensitivity to bending loss, and more likelihood of fiber defects during the manufacturing process [20,34,35].

### 2.2. Theoretical Derivations

When THz wave is injected into the fiber core, it will be absorbed by the analytes inside when passing through the MSF. The output light has a transmission intensity *I*(*f*) at frequency *f*. According to the modified Lambert–Beer law, *I*(*f*) can be expressed by the following formula [36,37]:

(1) $$I(f)=I_{0}(f)\exp[-s\alpha_{m}Lc],$$

where *I*_0_(*f*) refers to the input light intensity, *s* represents the relative sensitivity, *α_m_* represents the absorption coefficient of the analytes material, and *L* and *c* represent the thickness and density of the analytes material, respectively.

The absorption in light intensity by the analytes can be expressed as follows [38]:

(2) $$A=\log\frac{I}{I_{0}}=-s\alpha_{m}Lc.$$

Relative sensitivity, which represents the level of interaction between THz wave confined in the fiber core and the analytes to be measured, is considered one of the most important characteristics of the sensor. To measure the relative sensitivity, we need to determine the intensity of light directly interacting with the measured object, which can be calculated by the formula [36,38]:

(3) $$s=\frac{n_{a}}{n_{eff}}P_{R},$$

where *n_a_* represents the refractive indices of analytes, *n_eff_* represents the effective refractive index, and *P_R_* represents the power fraction. The power fraction represents the percentage of the optical power propagating in the fiber core to the total incident power, which can be calculated by the following formula [36,38]

(4) PR=∫analyteRe(ExHy−EyHx)dxdy∫totalRe(ExHy−EyHx)dxdy.

In the numerator, the field is integrated over fiber core area, and in the denominator, the field is integrated over the total fiber cross sectional area. *E_x_E_y_* and *H_x_H_y_* represent the components of the electric and magnetic fields in the x and y polarization directions, respectively.

EML is defined as the power loss caused by absorption of substrate materials and markers when the THz wave is transmitted in the waveguide, which is another important sensing property and can be calculated by the following formula [14,39]:

(5) $$\alpha_{eff}=\frac{{\left(\frac{\varepsilon_{0}}{\mu_{0}}\right)}^{\frac{1}{2}}\int_{mat}n_{mat}\alpha_{mat}{\left|E\right|}^{2}dA}{\left|\int_{mat}S_{z}dA\right|},$$

where *E* is the electric field, *S_z_* is Poynting vector in the z direction, *ε*_0_ and *μ*_0_ are the dielectric constant and permeability in vacuum, respectively. *n_mat_* and *α_mat_* denote the sum of refractive indices and absorption loss coefficients, respectively, of the substrate material and analyte markers in the core.

Due to the capability of fiber to bound light, a small portion of THz energy may escape from the fiber core, enter the cladding and eventually be absorbed by the PML. One characteristic parameter that represents the light-bounding capability of fiber is CL, which can be calculated by the formula [39]:

(6) αCL=4πλIm(neff),

where *λ* represents the wavelength, and Im(*n_eff_*) represents the imaginary part of the effective refractive index of the mode.

For MSF, due to the selection of arm thickness (*d* and *p*) and air hole size, the mode field area can change accordingly. The effective mode field area is defined as [40]:

(7) $$A_{eff}=\frac{{\left(\iint \left|E_{\left(x,y\right)}\right|{}^{2}dxdy\right)}^{2}}{\iint \left|E_{\left(x,y\right)}\right|{}^{4}dxdy}$$

where *E*_(*x*,*y*)_ represents the electric field intensity at point (*x*, *y*). After solving the electric field for MSF, designed with different structural parameters or analyte fillers, the effective mode field area can be calculated by carrying out the integrals in Equation (7).

NA is another important parameter of an optical fiber. It describes the ability of the optical fiber to collect THz waves, which affects THz wave injection efficiency. The study of NA plays a key role in light coupling between light source and fiber, or between fiber and fiber. Once the effective mode area is calculated by Equation (7), NA can be estimated by the following formula [18]:

(8) $$\mathrm{NA}\approx (1+\frac{\pi A_{eff}}{\lambda^{2}}{)}^{-\frac{1}{2}}=(1+\frac{\pi A_{eff}f^{2}}{c^{2}}{)}^{-\frac{1}{2}}$$

where *A_eff_* is effective mode area, *c* denotes the speed of light, and *λ* and *f* are wavelength and frequency of the transmitted light, respectively. From this formula, it is apparent that the NA of MSF is closely related to the wavelength, which is very different from conventional step-index fibers.

## 3. Modelling Results and Performance Analysis

Here, the analytes filled into the MSF are markers for DM (tablets made of albumin, glucose, glycated hemoglobin and triglyceride) and for CHD (triglyceride), respectively. The refractive index patterns of these markers with frequency are obtained from the literature [2,4,41]. To improve the sensing performance of the MSF, we use the control variable method to study the effect of fiber parameters, by changing the fiber size and cross-sectional configurations. After selecting the optimal parameters, markers of diseases are filled into the MSF core and fiber sensing performance is then characterized.

### 3.1. Optimization of MSF Constructing Parameters

**Figure 3:**
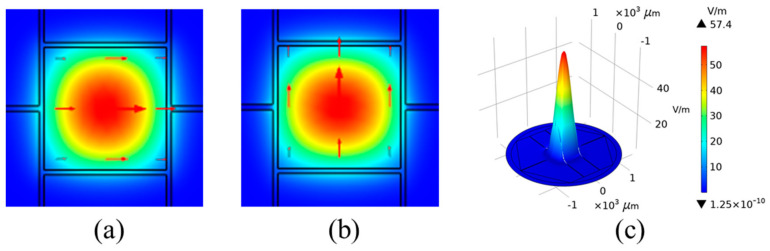
Mode field distribution of the proposed MSF with the specific structure parameters (*d* = 20 μm*, p =* 30 μm, *l* = 500 μm) for (**a**) x−polarization, (**b**) y polarization and (**c**) the three−dimensional diagram of the intensity distribution of the fundamental mode field.

Firstly, the mode field distribution of the proposed MSF, filled with normal levels of triglycerides as an instance, is shown in Figure 3. Specifically, in this case, the structure parameters are selected as *d* = 20 μm, *p* = 30 μm, and *l* = 500 μm, simulated at 1.0 THz for different polarizations. As can be seen, transmitted light is confined in the core, which provides a relatively large region of interaction between light and analytes. When operating at lower frequency bands, the simulation results show a larger mode field area and more electromagnetic energy loss. In contrast, when the MSF operates at higher frequency bands, the fundamental mode is well confined inside the core, and thus the mode field energy loss is reduced. The following work is to seek the optimal parameters for the designed MSF biosensor.

#### 3.1.1. Effect of Core Side Length l on MSF Sensor Performance

Firstly, the dependence of optical fiber performance on different side lengths *l* of the MSF core is discussed. Several important parameters of fiber performance are analyzed, including relative sensitivity, power fraction, EML, CL, NA, and effective area.

**Figure 4:**
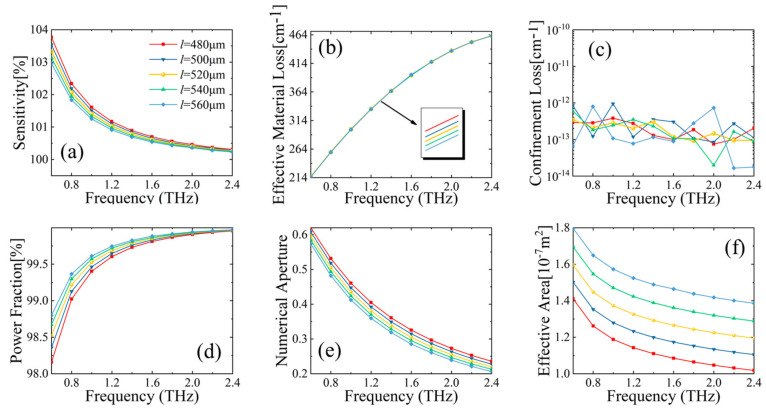
Behavior of (**a**) relative sensitivity, (**b**) EML, (**c**) CL, (**d**) power fraction, (**e**) NA, and (**f**) effective area, with respect to frequency for different core side length *l* of MSF.

Figure 4 shows the performance parameters of the designed MSF when side length *l* varies over 480~560 µm, with a transmission frequency range of 0.6~2.4 THz. From Figure 4a, it can be seen that the relative sensitivity decreases with increasing transmission frequency. This is because of an effective index difference between the core and cladding. As transmission frequency increases, the index difference gradually increases and the THz wave is better confined within the core. Thus, as the effective index of the fundamental mode increases with frequency, the relative sensitivity decreases as related by Equation (3). In Figure 4b, the dependence of EML on different core side lengths *l* is plotted over frequency. As shown, EML increases monotonically with frequency, and EML decreases slightly with the increase of core side length *l*. From Equation (5), the effective material loss of MSF is positively correlated with the material absorption coefficient. The CL of the proposed MSF with frequency is plotted in Figure 4c, which shows that CL varies very little in the tested frequency range. The CL basically stabilizes around 10^−13^ cm^−1^ over the range of 1.6~2.4 THz for a core length of 520 µm, which proves that the designed structure has a good light confining effect for all frequencies. In Figure 4d, the response of the power fraction with the increment of frequency is depicted. Values of the power fraction first rapidly increase within a frequency range of 0.6~1.2 THz, and then slowly increase over the 1.2~2.4 THz range. The reason is that at lower frequencies than 1.2 THz, the interaction of light and analytes increases with frequency; however, at frequencies higher than 1.2 THz, a vast majority of optical power is already concentrated in the inner core and therefore, the power fraction changes slowly with frequency. Figure 4e depicts the NA change as a function of frequency, which shows a monotonically decreasing trend. In this case, NA also decreases with the increase of core side length *l*, indicating that light collection is better with a smaller core aperture. In Figure 4f, effective areas as a function of frequency for different core sizes are depicted. From the curves, we can tell that the effective area monotonically decreases with frequency. With increasing frequencies, light is being more tightly bound to the core region, and as a result, the effective area decreases. Figure 4f also tells us that increasing core size enlarges the effective area. The reason is that, when the core is larger, the mode power can be more spread within the porous core, which consequently leads to a larger effective area. To ensure the overall transmission effectiveness of the fiber, the performance of the fiber parameters as a function of aperture diameter needs to be considered in a comprehensive manner. The core side length *l* = 500 µm is finally determined as one of the optimal fiber structure parameters.

#### 3.1.2. Effect of Vertical Support Arm Thickness d on MSF Sensor Performance

**Figure 5:**
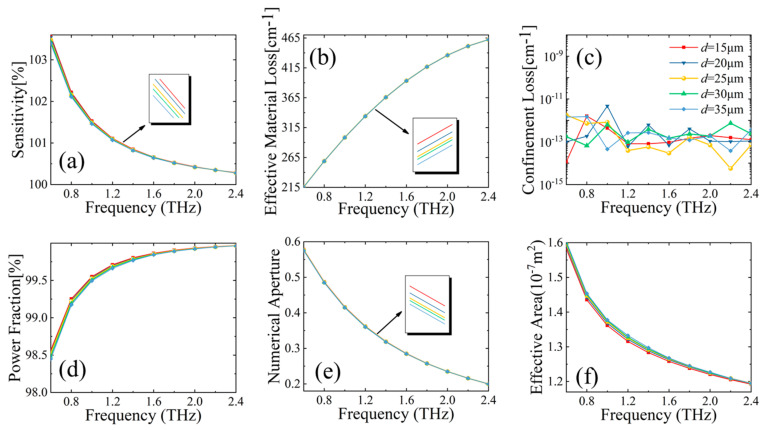
Behavior of (**a**) relative sensitivity, (**b**) EML, (**c**) CL, (**d**) power fraction, (**e**) NA, and (**f**) effective area, with respect to frequency for different vertical arm thickness *d* of MSF.

Secondly, we investigate the effect of vertical support arm thickness *d* on fiber performance. Once again, with different values of *d*, we conduct a comparative analysis, shown in Figure 5, of the six important performance factors: relative sensitivity, power fraction, EML, CL, NA, and effective area.

The results in Figure 5a are similar to those in Figure 4a: relative sensitivity decreases with the increase of transmission frequency. Figure 5b shows the change of EML over frequency. The impact from changing supporting arm thickness *d* is minimal compared to the effect of frequency change. As the frequency increases, more light leaks into the background material, resulting in higher losses. The CL of the proposed MSF is plotted in Figure 5c over frequency. As observed, for all thickness *d* values under consideration, CL values remain within a relatively flat range around 10^−13^ cm^−1^. Figure 5d shows the change of power fraction over frequency. With increasing frequencies, more and more power will be confined within the region where analytes reside. Figure 5e clearly demonstrates a monotonically decreasing trend of NA vs. frequency. In this case, NA also decreases with the increase of vertical arm thickness *d*. Figure 5f shows that effective area is a decreasing function over frequency from 0.6 THz to 2.4 THz. With increasing frequencies, light is being more tightly bound to the core, and as a result, the effective area decreases. With decreasing vertical arm thickness *d*, the effective area also reduces its value. After weighing the advantages and disadvantages of increasing the vertical support arm on the performance parameters of the MSF, the thickness of the vertical support arm is determined as *d* = 25µm.

#### 3.1.3. Effect of Horizontal Support Arm Thickness p on MSF Sensor Performance

**Figure 6:**
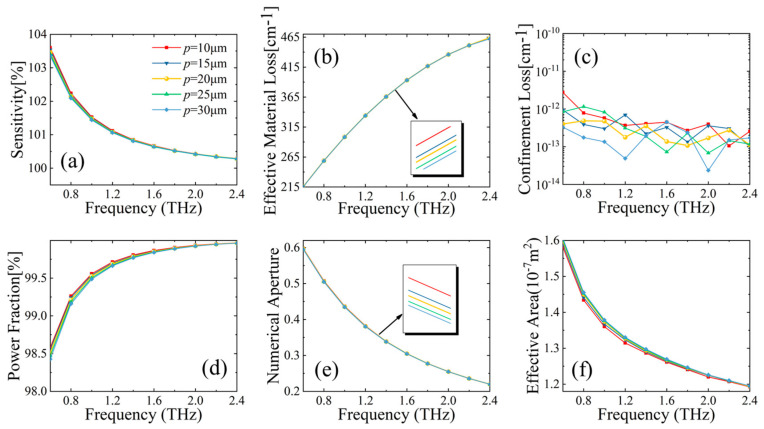
Behavior of (**a**) relative sensitivity, (**b**) EML, (**c**) CL, (**d**) power fraction, (**e**) NA, and (**f**) effective area, with respect to frequency for different horizontal arm thickness *p* of MSF.

Characteristics of fiber performance by varying the thickness *p* of horizontal support arms are shown in Figure 6. It can be observed that, variation trends of the six simulated parameters (relative sensitivity, power fraction, EML, CL, NA, and effective area) over frequency are similar to those in Figure 5. It is worth noting that, at the chosen optimal design (with a core diameter of 520 µm, horizontal support arm thickness 20 µm, and vertical support arm thickness 25 µm), the MSF biosensor demonstrates a low CL of about 10^−13^ cm^−1^, an ultra-high sensitivity of 100.35%, and a small effective area of about 1.23 × 10^−7^ m^2^ at 2.2 THz.

### 3.2. Exploring a High-Sensitivity MSF Sensor for Detecting DM and CHD 

Based on the MSF design, we simulate situations where different concentrations of DM and CHD disease markers are added to the core region of the designed biosensor. In particular, we analyze the differences in sensitivity, EML, and other parameters of the sensor, when marker concentrations in patient blood are at normal, high, and critically high level, respectively.

#### 3.2.1. Detection of DM Markers by the MSF Sensor

Plasma samples from diabetic patients and a normal group are filled into core of the proposed MSF, and the differences in properties presented by MSF are compared. The evolution of diabetes is often accompanied by elevated concentrations of glucose, glycosylated proteins, and various regulatory hormones in the blood [2]; therefore, the refractive indices as well as absorption coefficients of markers in the blood of diabetic patients are generally higher than those of the normal group [2,42].

**Figure 7:**
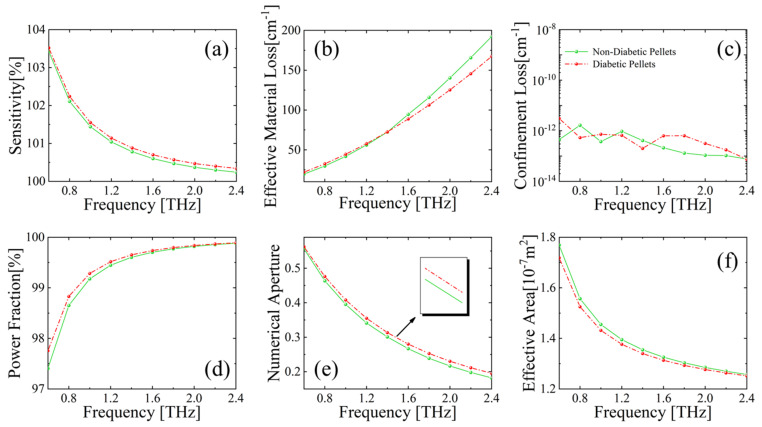
Behavior of (**a**) relative sensitivity, (**b**) EML, (**c**) CL, (**d**) power fraction, (**e**) NA, and (**f**) effective area, with respect to frequency for MSF core filled with DM marker.

Figure 7 shows the sensing performance of the proposed MSF biosensor, with different levels of diabetic pellets (DM markers), where the serum samples made into diabetic tablets of diabetic patients had glucose levels of 6.51 mmol/L, triglyceride levels of 1.69 mmol/L, and glycosylated hemoglobin levels of 11.0%; the serum samples of normal volunteers had glucose levels of 4.34 mmol/L, triglyceride levels of 0.82 mmol/L, and glycosylated hemoglobin levels of 4.8%. Refractive indices of diabetic patient bloods, due to DM markers, are higher than the normal human blood. A polynomial fit analysis of the experimental data extracted from Reference [2] shows that the refractive indices of diabetic markers and non-diabetic markers follow the given relations over frequencies: *n_d_* = 1.91 − 0.11*f* + 0.02*f*^2^, *n_nd_* = 1.84 − 0.19*f* + 0.02*f*^2^, respectively, and for both relations, the regression parameters R^2^ are greater than 0.996. Therefore, according to Equation (3), relative sensitivity for high the DM marker case is higher than the normal case, as shown in Figure 7a. EML, as expressed by Equation (5), is proportional to the material absorption coefficient. According to the results in Reference [2], it is known that the absorption coefficients of diabetic markers and non-diabetic markers satisfy the following relations: *α_d_* = −1.72 + 28.12*f* + 17.54*f*^2^ and *α_nd_* = 1.2 + 14.63*f* + 25.57*f*^2^, respectively. The variations of EML with frequency, as shown in Figure 7b, correspond well to the absorption coefficients of different DM marker concentrations. Figure 7c shows the trend of CL with frequency for the MSF sensor. As can be observed, CL values markers are basically distributed around 10^−13^ cm^−1^ at 2.0~2.4 THz, which are relatively small compared with previously reported work [18,40,43,44]. This can be understood in that the designed MSF has a suspended hollow-core structure, which helps bound the THz light in the core and thus reduces the CL. All other trends in Figure 7 are quite similar to Figure 4, Figure 5 and Figure 6, as discussed before.

#### 3.2.2. Detection of CHD Markers by the MSF Sensor

**Figure 8:**
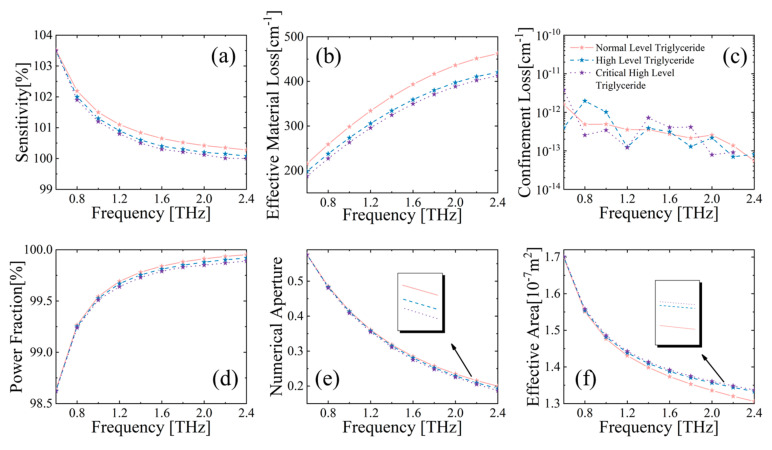
Behavior of (**a**) relative sensitivity, (**b**) EML, (**c**) CL, (**d**) power fraction, (**e**) NA, and (**f**) effective area, with respect to frequency for MSF core filled with CHD marker.

One of the most dangerous causes of CHD is abnormal blood lipids in humans. Therefore, rapid and accurate calibration of triglyceride levels in human serum is crucial for clinical diagnosis of this disease [4]. The performance of the MSF biosensor filled with CHD markers is shown in Figure 8. As can be observed in Figure 8a, the relative sensitivity of MSF decreases with increasing concentrations of triglyceride in the serum. This is because higher triglyceride concentrations lead to lower refractive indices of the serum solutions, and from Equation (3), the relative sensitivity of MSF will decrease. According to the data reported in Reference [4], the absorption coefficients of normal concentration, high concentration, and critical high concentration markers satisfy the following relations: *α_n_* = 50.8 + 296*f* − 52.09*f*^2^, *α_h_* = 48.81 + 296.11*f* − 47.81*f*^2^, *α_ch_* = 38.55 + 295.57*f* − 47.14*f*^2^, respectively. Figure 8b shows that EML decreases with increasing concentrations of the coronary marker, which is mainly attributed to the change in absorption coefficients with marker concentrations. It is noteworthy that the EML differs dramatically between normal levels of triglycerides and high levels of triglycerides, which is beneficial for the accuracy of disease diagnosis. The trends of other parameters of the MSF with frequency are also similar to Figure 7.

In order to compare the operational performance of the proposed methodology, we consider various biochemical materials, as reported in the literature, and simulate the characteristic parameters using our proposed MSF. As shown in Table 1, comparing with results from previously reported sensors, our MSF sensor shows advantages over the main parameters (relative sensitivity, CL and EML). This shows that the selection of appropriate substrate materials, and a well-designed structure of suspended hollow core can greatly improve the sensing performance.

## 4. Feasibility of Preparation for the Designed MSF Biosensor

The combination of 3D printing and extrusion technologies enables rapid and precise fabrication of MSFs, which typically involves extruding a molten cyclic olefin polymer through a 3D printing nozzle and stretching it directly to the size required for THz sensing [20,35]. Compared with other traditional techniques for fiber preparation, this technique simplifies the fiber prefabrication and enables precise manufacturing of MSF structures with large duty cycles [35]. In recent years, by continuously optimizing MSF end face design and 3D printing fabrication schemes, the prepared fibers have achieved low-loss operating performance over a wide frequency range [34]. Therefore, 3D printing and extrusion technologies show great promises for high-quality, flexible MSF preparations in the future.

**Figure 9:**
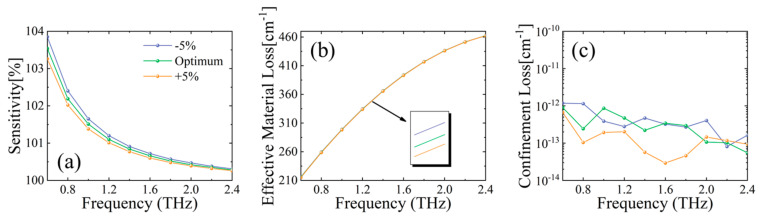
Behavior of (**a**) relative sensitivity, (**b**) EML, and (**c**) CL for a 5% change in fiber geometries.

In order to quantitatively study manufacturing effects, fabrication tolerance (FT) analysis is carried out on the proposed MSF by altering geometric parameters by 5% from the optimum (i.e., varying all parameters together in scale to 95%, or 105%). For this analysis, we use normal levels of triglycerides filled in the core as an example. Overall, the performance of the optical fiber, as shown in Figure 9, does not change dramatically over the 5% FT. In Figure 9a, the maximum change in relative sensitivity is less than 1%. Similarly, in Figure 9b, EML change of the MSF is almost negligible over all frequencies, thanks to the strong THz-bounding capability given by the applied suspended hollow-core structure. When the overall size of the fiber increases by 5%, CL, as shown in Figure 9c, decreases to values around 10^−13^ cm^−1^ in the 0.8~2.4 THz frequency band. When the overall size is reduced by 5% from optimum, CL remains largely the same values as the optimum.

## 5. Conclusions

In this paper, an ultra-high-sensitivity MSF biosensor is proposed for DM and CHD disease marker detection using THz waves. Using a full-vectorial finite element method, we analyze optical sensing characteristics of the proposed Zeonex-based MSF sensor. The results show that our MSF biosensor, when filled with DM and CHD biochemical markers, performs well with optimal design parameters (specific values: *l* = 520 μm, *d* = 25 μm, *p* = 20 μm), with a demonstrated ultra-high relative sensitivity of 100.35% and a low coupling loss of only around 10^−13^ cm^−1^, both estimated at 2.2 THz, showing better results compared to sensors reported before [45,46]. The maximum numerical aperture reaches 0.58 at 0.6 THz, which also reflects a significant advantage of using the suspended hollow-core design in MSF. With a fabrication tolerance of 5%, our designed MSF biosensor deviates less than 1% in performance compared to the optimum. In addition, the MSF in this study is structurally simple and convenient to fabricate using existing 3D printing and extrusion technologies. With this fiber sensor design, implemented in a THz time-domain spectroscopy system, we believe that it can be conveniently adopted for many potential biomedical marker detection and quick diagnosis applications.

## Figures and Tables

**Table 1:** Comparison of sensing operation performance over various biochemical materials.

Reference	Biological Materials	Sensitivity [%]	CL [cm^−1^]	EML [cm^−1^]
[9]	Benzene	89.00	1.15 × 10^−9^	0.0280
This Work		99.76	1.87 × 10^−13^	0.0040
[16]	Ethanol	85.70	1.70 × 10^−7^	-
This Work		99.73	2.74 × 10^−13^	0.0042
[28]	Glucose	47.59	-	-
This Work		99.71	3.84 × 10^−13^	0.0043
[28]	Human Mucosa	47.31	-	-
This Work		99.68	1.94 × 10^−13^	0.0044
[29]	Red Blood Cells	94.38	2.75 × 10^−13^	-
This Work		99.80	1.81 × 10^−13^	0.0036

## Data Availability

Not applicable.
